# Health technology assessment to support health benefits package design: a systematic review of economic evaluation evidence in Zambia

**DOI:** 10.1186/s12913-024-11914-z

**Published:** 2024-11-18

**Authors:** Warren Mukelabai Simangolwa, Kaymarlin Govender, Josue Mbonigaba

**Affiliations:** 1 Health Economics and HIV/AIDs Research Division, University of KwaZulu Natal, Durban, South Africa; 2 College of Law and Management Sciences, University of KwaZulu Natal, Durban, South Africa; 3 Centre for Health Economics Financing and Technology Assessment, Patient and Citizen Involvement in Health, 3739 Kwacha Road, P.O Box 310159, Olympia, Lusaka, Zambia

**Keywords:** Health technology assessment, Health benefits package, Economic evaluation, Cost-effectiveness analysis, Cost, Systematic review

## Abstract

**Background:**

Health technology assessment uses explicit economic evaluation evidence to support health benefits package design. However, the limited availability of technical expertise, data, and methods has restricted the production of economic evaluation evidence in low- and middle-income countries. Zambia has initiated a roadmap to support its policy of reviewing and implementing its national benefits package. This study characterises economic evaluation evidence to support this process's evidence mapping, synthesis, and appraisal stages.

**Methods:**

This systematic review applies deductive analysis and the preferred reporting items for systematic review and meta-analyses. Four databases were searched to identify studies from 1993 that coincided with Zambia's health benefits package reform.

**Results:**

A total of 61 studies met the inclusion criteria. Most of the studies were first authored by nonlocal authors, and the number of local-based authors in each study was low. Almost all funding for economic evaluation research was not local, and only a few studies sought local ethical clearance to conduct research. Infectious diseases were the highest disease control priority for the studies, with HIV research having the highest output. Most of the studies were cost-effectiveness studies that utilised trial-based data and a combination of program, published, and unpublished data for analysis. The studies generally utilised direct cost and applied the ingredient-based costing approach. Natural units were predominantly used for outcomes alongside DALYs. Most studies reported using a 3% discount rate for both costs and outcomes, with only a few reporting methods for sensitivity analysis.

**Conclusion:**

Economic evaluation evidence in Zambia has increased, revealing limited local research leadership, methodological inconsistencies, and a focus on infectious diseases. These findings are crucial for revising Zambia's benefits package and may guide researchers and decision-makers in improving the transparency and quality of future research.

**Supplementary Information:**

The online version contains supplementary material available at 10.1186/s12913-024-11914-z.

## Introduction

The World Health Assembly passed Resolution WHA 67.23, urging member states to utilise health technology assessment (HTA) to support universal health coverage policy decisions, including benefits package design [1]. The barriers to producing HTA in low- and middle-income countries (LMICs) include limited local budgets, data availability, methods, and in-country technical capacity [1–5]. HTA is a multidisciplinary approach that applies explicit methods, including economic evaluation, to determine the value of health technology for decision-making [6]. While the value established by HTA applies to a single health technology, economic evaluation output contributes to the prioritisation evidence necessary to support benefits package design and the inclusion of new health technology in its revision [7, 8]. In the 2021 global survey on HTA and health benefit packages, fewer than 19% of LMICs reported having available methods for conducting economic evaluations [4]. Only one country-specific economic evaluation methodological guideline was observed in Sub-Saharan Africa, with methodological gaps and contradictions common in all thirteen guidelines identified in LMICs [9]. Without country-specific guidelines, researchers have alternatively used international disease-specific guidelines, albeit with contextual challenges in appropriately estimating costs and effectiveness [10].

Methodological studies on economic evaluations in LMICs have identified gaps, including limiting cost measurement to either a top-down or bottom-up approach, neglecting the statistical analysis of cost data and limiting the use of routine cost data, probabilistic sensitivity analysis, quality of life assessments and cost-effectiveness thresholds [10–13]. A global bibliographic analysis of 2,844 economic evaluation studies revealed a disproportionately large number of publications, authors, and research funding from high-income countries, as well as research dominance in HIV/AIDS, maternal and neonatal care, and malaria [11]. Country-specific economic evaluation review studies in India, Vietnam, Bangladesh [14–16], and sub-Saharan countries [17–21] reinforce these findings and further highlight the lack of local technical capacity and consistent methods [17–19].

The government of Zambia proposes to establish a formal HTA institution and use HTA evidence to support health benefits package design and implementation [22, 23]. However, limited funding, networking, and in-country technical expertise have posed challenges to the institutionalisation, production, and use of HTA [2, 23, 24]. To mitigate this, Zambia has developed a roadmap to design and implement the National Health Care Package to support its aspiration towards universal health coverage [23]. The National Health Care Package was established in 2012 and is an overarching package containing interventions at all levels of care. It includes the national health insurance package, initiated in 2019, containing hospital-level interventions [23]. The government proposes that economic evaluation data be used in the design of the national health care package and that HTA be applied to its revision when including new health technology [23]. The government also advocates using HTA to revise the national health insurance benefits package to include primary health technologies [25].

The Zambia benefits package revision roadmap has identified critical HTA technical expertise and evidence requirements for all three phases: foundational, evidence and implementation. According to the benefits package roadmap [23], the foundational phase (landscape analysis, process development, and intervention mapping) identifies the scope, criteria, and data needs for the benefits package revision process. In contrast, the evidence phase involves conducting evidence collection, synthesis and appraisal. The implementation phase is reserved for planning and implementation, monitoring and evaluation, political and public involvement, decision-making and considerations for future revisions.

However, within the evidence phase of the roadmap, the conduct and quantity of economic evaluation evidence to support intervention mapping, data collection, and synthesis to guide decision-making for evidence appraisal have not been curated [23]. This systematic review characterises health economic evaluation evidence in Zambia to map the available quantity, methods, and practices to support decision-making for these critical stages of the benefits package revision process.

## Methods

### Design

This study is reported using the Preferred Reporting Items for Systematic Reviews and Meta-Analyses and follows a framework proposed by Van Mast et al. [26, 27]. Supplementary File 1 itemises this article’s content with the PRISMA checklist.

### Eligibility criteria

Original Zambia-specific or multi-country studies were included if they produced economic evaluation results for Zambia. The inclusion criteria were restricted to English language studies published between 1993 and 2023 to coincide with early initiatives on health benefits packages. Studies that evaluated cost or health outcomes only or both but did not produce an economic evaluation outcome were excluded. Studies whose economic evaluation evidence did not directly impact the health sector, systematic reviews and meta-analyses, studies not peer-reviewed, abstracts and conference papers were also excluded.

### Information sources and searches

We searched PubMed, Scopus, the NHS Economic Evaluation Database, the Tufts Medical Center Cost-Effectiveness Analysis Registry and the results of the first 200 studies in Google Scholar. We used medical subject headings terms with keywords and variations in economic evaluation, cost-utility, cost benefits, cost-effectiveness and Zambia; Supplementary File 2 contains the search terms and a PubMed electronic search strategy.

### Selection of sources of evidence

All the authors, WS, JM and KG, screened the same 15 studies, discussed the results and modified the screening and data extraction form. In the first screening stage, two reviewers, WS and JM, independently screened the prospective publications by title and abstract and applied the eligibility criteria for study selection. In the second stage, WS and JM retrieved and further screened the full-text articles for inclusion eligibility. All disagreements were resolved through discussion and consensus by WS, JM and KG.

### Data chartering process and data items

A data chartering form was designed for this study in Microsoft Excel. It contains a selection of background characteristics described by Van Mastigt and items from the Drummond et al. checklist [26, 28]. Data from article characteristics included the publication year, article URL, article title, sources of funding, first author, first author's country and institution, number of authors, locally-based authors, local-only authors, and local institutions identified. Other characteristics were the journal of publication, ethical clearance status, research setting, intervention type and cost-effectiveness threshold used. To extract data for the first author's country, we used the country with which the first author’s institution was affiliated. In cases where an author had multiple affiliations, a combined classification of the countries was used, i.e., Zambia/USA. The study also applied the WHO Universal Health Coverage compendium of health services to each study's health technology to map details on the group, subgroup, intervention category, intervention and action categories [29]. Background characteristics of the final version of the data chartering form are included in Supplementary File 3.

### Synthesis of Results

All included studies were analysed deductively. We used Microsoft Excel to derive counts, percentages and descriptive statistics. All synthesised data are presented in tables and graphs. We did not perform risk of bias and quality assessments in this study.

## Results

**Fig. 1:**
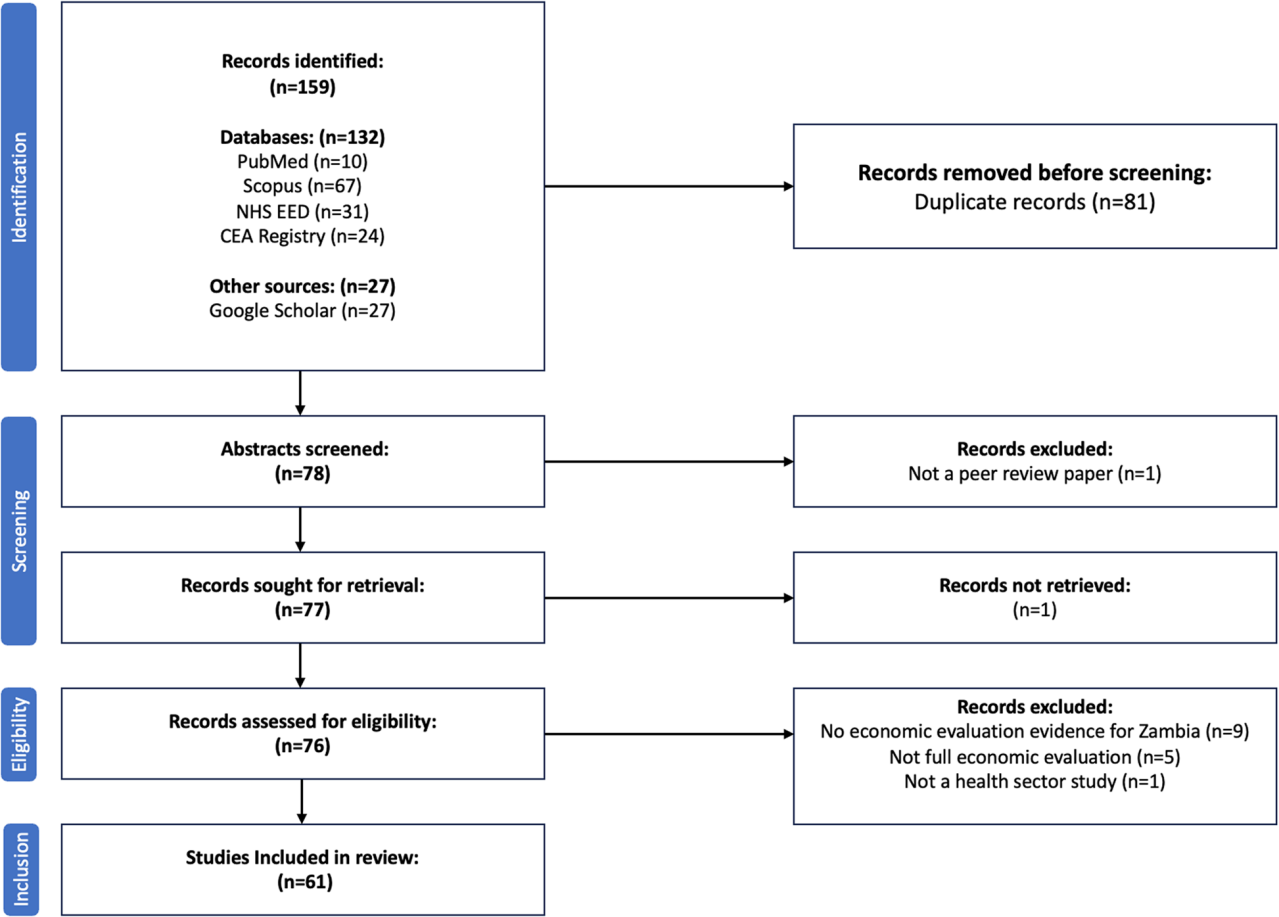
Study selection process flowchart

The study selection process is summarised in the study flow chart in Fig. 1. A total of 159 studies were identified, of which 81 duplicate studies were removed, leaving 78 studies for screening. An additional study was excluded based on the title and abstract. Of the 77 remaining studies, one could not be retrieved, and 15 did not match the selection criteria and were removed after full screening. As a result, 61 papers were included in the review. The included studies were imported into Zotero-6.0.13 for reference management.

### Background characteristics of the included studies.

**Fig. 2:**
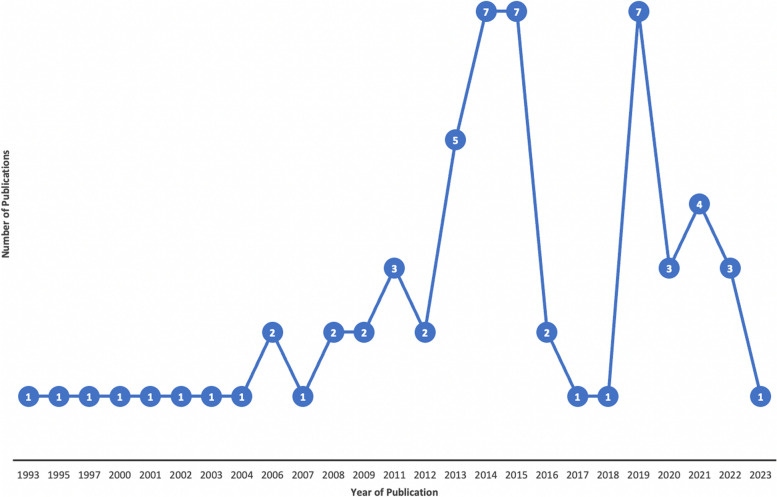
Economic evaluation studies publication timeline in Zambia (1993–2023)

Figure 2 and Table 1 show the characteristics of the included studies. Before 2004, there was no more than one publication produced each year. The number of included publications more than doubled from 2012 onwards compared to the period before (1993–2011), increasing from 18 to 43 [30–90].

**Table 1:** Funding and author background characteristics of included studies

Characteristics	Frequency	Percentage
Research Funders		
Bill and Melinda Gates Foundation	16	17%
United States Agency for International Development	9	9%
United States National Institutes of Health	6	6%
World Health Organisation	6	6%
Department for International Development	8	8%
United States President's Emergency Plan For AIDS Relief	4	4%
United States Center for Disease Control and Prevention	4	4%
European Union	3	3%
Others: With Two or Less Funding Support	42	44%
No Funding	3	3%
Funder Role in Funded Research		
No Stated	8	14%
No Role	50	86%
Country of the First Author		
USA	27	44%
UK	12	20%
Zambia	6	10%
Zambia/USA	4	7%
Netherlands	3	5%
Japan	2	3%
Others: One Publication for Each First Author	7	11%
(Zambia/UK, China, Ireland, QATAR, Norway, South Africa, Israel)		
Institution of First Authors		
London School of Hygiene and Tropical Medicine	7	11%
Centre for Infectious Diseases Research in Zambia	4	7%
Johns Hopkins University	4	7%
National Malaria Control Centre at the Ministry of Health	3	5%
Others: Institutions with two first-authors and less	43	70%
Number of Authors		
Number of Authors in all Publications	563	
Number of Local Affiliated Authors	144	
Number of Local-only Authors	120	
Affiliations for Local-only Authors		
Ministry of Health	45	38%
University of Zambia	18	15%
Centre for Infectious Disease Research in Zambia	17	14%
PATH Malaria Control and Elimination Partnership in Africa	9	8%
Macha Research Trust and Macha Mission Hospital	5	4%
Others: 26 affiliations for authors appearing less than five	26	22%

The Bill and Melinda Gates Foundation and the United States Agency for International Development provided 17% and 9% of the funding for economic evaluation research, respectively. The Department for International Development and the World Health Organisation were the third and fourth top research funders, with funding of 8% and 6%, respectively. The funders for each study ranged from 0 to 5 in each included study. Only one study was funded by a Zambia-based organisation, and in three studies, the researchers received no funding. In 82% of the studies, the researchers stated that funders had no role in the study design, data collection and analysis, decision to publish, or manuscript preparation. However, 14% of the funded studies did not specify whether the funder influenced the research process beyond providing financial support..

The United States of America (USA) had the highest number of first authors at 44%, followed by the United Kingdom (UK) at 20% and Zambia at 10%. An additional 7% of the first authors were from the Zambia/USA author combination category. First authors from the Netherlands and Japan were at 5% and 3%, respectively. Zambia/UK, China, Ireland, Qatar, Norway, South Africa and Israel each had a single first author. Regarding institutions of affiliation, more first authors were affiliated with the London School of Hygiene and Tropical Medicine, which at 11%. The Center for Infectious Diseases in Zambia and Johns Hopkins University had 7% of the first authorship each. Seventy per cent of first-author institutions appeared only once or twice. Thus, while the USA has many first authors, they represented diverse institutions.

As shown in Table 1, a total of 563 authors authored all the 61 studies. Each study's minimum and maximum authorship ranged from 1 to 52 authors. A total of 120 authors were exclusively affiliated locally, with each study comprising between 0 and 8 authors associated with local institutions. The Ministry of Health had the highest number of authors within Zambia at 38%, followed by the University of Zambia at 18% and the Centre for Infectious Disease Research in Zambia at 14%. The National Malaria Control Centre within the Ministry of Health contributed 18 authors. Authors from the Zambia AIDS-Related Tuberculosis constituted 12 of the 18 Authors affiliated with the University of Zambia.

All publication journals for economic evaluations in Zambia were international (see Table 2). PLOS One published the highest number of publications at 16%. The AIDS journals accounted for 11%, whereas the Malaria and Lancet Global Health journals published 10% and 7% of the studies, respectively. A total of 27 studies, representing 44.3% of the included studies, did not state if they had received ethical authorisation or exemptions to carry out their studies. In 18% of the studies, the authors explicitly indicated that they had not received ethical clearance. Author justifications were that the study did not involve human subjects, did not require ethical approval, was not applicable, and patient records were not collected or reviewed. Eighteen per cent (18%) of the included studies received ethical clearance in Zambia only, 14.8% received clearance from Zambia and additional countries, and 4.9% received ethical clearance from another country only. Of the 20 studies that received ethical clearance in Zambia, one obtained two clearances, resulting in 21 clearances. The University of Zambia Biomedical Research Ethics Committee granted the highest number of ethical clearances, accounting for 71.4% of all approvals. The Excellence in Research Ethics and Science, along with the Zambia National Health Research Authority, accounted for 14.3% and 9.5% of the total 21 ethical clearances obtained in Zambia.

**Table 2:** Journal, study setting and ethics background characteristics of included studies

Characteristics	Frequency	Percentage
Journal of Publication		
PLOS One	10	16%
AIDS	7	11%
Cost Effectiveness and Resource Allocation	7	11%
Malaria Journal	6	10%
Lancet Glob Health	4	7%
Tropical Medicine and International Health	3	5%
International Journal of Tuberculosis and Lung Diseases	2	3%
PLOS Medicine	2	3%
Global Health: Science and Practice	2	3%
Others: 18 Journals published in only once	18	30%
Research Setting		
Zambia	46	75%
Zambia and Others Countries	15	25%
Research Setting by Province		
Southern	22	27%
Lusaka	21	26%
Eastern	11	14%
Copperbelt	11	14%
Central	7	9%
Luapula	3	4%
Northern	2	2%
Western	2	2%
Muchinga	2	2%
North-Western	0	0%
Ethical Clearance		
Zambia only	9	14.8%
Zambia and Other country(s)	12	19.7%
Other Country	3	4.9%
None/not specified	37	60.7%
Local Institutional Review Boards Identified		
University of Zambia Biomedical Research Ethical Committee	14	66.7%
Excellence in Research Ethics and Science	3	14.3%
Tropical Disease Research Centre	2	9.5%
Zambia National Health Research Authority	2	9.5%

The research setting was dominated by studies undertaken in Zambia at 75%, whereas 25% wereere studies conducted in Zambia and elsewhere. Across Zambia, the southern province had study sites, at 27%, Lusaka had the second most, at 26%. As shown in Table 2, Luapula, Northern, Western, and Muchinga provinces accounted for only 11% of the study sites combined, with the North-Western province having no study site for all included economic evaluations.

### Methods and analysis of evidence characteristics of the included studies

The type of economic evaluation was explicitly stated in all studies. As shown in Table 3, Cost-effectiveness analysis studies accounted for the most prominent type at 93%, whereas cost–benefit analysis was used in only 7%. Cost-effectiveness analysis refers to all studies using a non-monetary measure of benefit, including generic measures such as quality-adjusted life years (QALYs) and disability-adjusted life years (DALYs). Using the World Health Organisation’s compendium, studies on infectious diseases accounted for 52.5%, whereas those focused on reproductive and sexual health comprised 37.7%. Growth, development, ageing, non-communicable diseases, mental health and foundations of care accounted for 9.8% of the included studies. Within the intervention category, 32.8% of the economic evaluation’s health technology was on HIV, followed by malaria at 19.7%, antenatal care at 11.5% and tuberculosis at 8.2%. The other intervention categories are shown in Table 3, and across all 61 included studies, 44.3% focused on programs, and 29.5% focused on drugs. Laboratory, diagnostic, and screening health technologies constituted 19.7%, while other categories, including medical devices, products, and procedures, accounted for 6.6%. The category ‘programs’ included non-pharmacologic health technologies involving health promotion and prevention, i.e., the cost-effectiveness of the strategy for HIV testing and counselling for couples together [76].

**Table 3:** Methods characteristics of the included studies

Characteristics	Frequency	Percentage
Type of economic analysis		
Cost benefits	4	66%
Cost effectiveness	57	93.4%
Disease Control Priority		
Infectious diseases	32	52.5%
Reproductive and sexual health	23	37.7%
Growth, development and ageing	3	4.9%
Non-communicable diseases and mental health	2	3.3%
Foundations of care	1	1.6%
Intervention Category		
HIV	20	32.8%
Malaria	12	19.7%
Antenatal care	7	11.5%
Tuberculosis	5	8.2%
Labour and childbirth care	4	6.6%
Undernutrition	3	4.9%
Postnatal care	2	3.3%
Contraception and family planning	2	3.3%
Others: 6 intervention categories ( sexual violence, Cholera	6	9.8%
Breast cancer, Meningitis, diarrhoeal, Eye conditions)		
Health Technology		
Program	27	44.3%
Drugs/ Pharmaceuticals	18	29.5%
Laboratory and Diagnostic/Screening	12	19.7%
Other e.g. medical device, product or procedure	4	6.6%
Perspective of analysis		
Provider/Health System	26	42.6%
Societal	5	8.2%
Payer	2	3.3%
Donor	1	1.6%
Provider and Donor	1	1.6%
Provider and Societal	2	3.3%
Not specified	24	39.3%
Model used		
Decision tree	4	6.6%
Markov	3	4.9%
Markov and Decision tree	3	4.9%
Others	10	16.4%
Not specified	41	67.2%
Data sources		
Trial-based	15	24.6%
Before and after design	1	1.6%
program-based	6	9.8%
Published and unpublished	13	21.3%
Program and published and/or unpublished	22	36.1%
Trial and published and/or unpublished	4	6.6%

A total of 26 studies, representing 42.6%, applied the health provider perspective, also referred to as the health system, health care system, and government perspective. Thirty-nine-point three per cent, of the studies, did not specify the perspective adopted in their analysis. Other perspectives used were societal at 8.2%, payer at 3.3%and a combination of provider and societal perspectives at 3.3%. The donor, and combination of donor and payer perspectives were utilised in only one study each.

Forty-one studies, representing 67.2%, did not specify the type of model used in their analysis. Among the remaining 20 publications, the decision tree, Markov, and a combination of decision tree and Markov methods accounted for 6.6%, 4.9% and 4.9%, respectively. Trial-based data were used more often across studies, accounting for 15 of the 61 included publications, followed by program-based studies at 9.8%. Table 3 shows that trial-based data sources were the primary study data source. Among the included studies, 36.1% utilised a combination of three types of data sources: program data, published, and unpublished. Additionally, 6.6% of the studies incorporated trial-based, published, and unpublished data sources.

Direct costs were used in 75.4% of the studies, with the remaining using a combination of direct and indirect costs (see Table 4). Direct costs refer to the resources associated with the health technology (including non-health direct costs such as out-of-pocket payments). In contrast, indirect costs are related to its impacts, such as productivity losses, i.e. direct and indirect costs identified in the included paper by Ryan, Máirín and others [41]. The ingredient-based approach, which was also called micro-costing, bottom-up, and activity-based, was applied to generate costs in 21 studies. In contrast, only two studies employed the top-down approach, and one study used the top-down and bottom-up approaches. A disproportionally large number of included studies, 37, needed to specify the costing approach used.

**Table 4:** Results characteristics of the included studies

Characteristics	Frequency	Percentage
Costs used		
Direct	46	75.4%
Direct and Indirect	15	24.6%
Costing approach		
Micro/ingredients/bottom-up/Activity-based	21	34.4%
Top-down	2	3.3%
Top-down and bottom- up	1	1.6%
Not Specified	37	60.7%
Outcome measures		
Natural Units	31	50.8%
DALY	9	14.8%
QALY	8	13.1%
DALY and natural units	11	18.0%
QALY and natural units	1	1.6%
DALY, QALY and natural Units	1	1.6%
Time horizon		
Less than 1 year	19	31.1%
1 to 5 years	19	31.1%
6 to 10 years	5	8.2%
11 to 15 years	2	3.3%
16 to 20 years	2	3.3%
Lifetime	6	9.8%
Not specified	8	13.1%
Discount rate		
3% Costs only	13	21.3%
3% Benefits only	7	11.5%
3% Costs and benefits	20	32.8%
5% Costs	2	3.3%
5% Benefits	1	1.6%
Not specified	18	29.5%
Sensitivity analysis		
Univariate	5	8.2%
Multivariate	3	4.9%
Univariate and multivariate	4	6.6%
Probabilistic sensitivity analysis	10	16.4%
Probabilistic sensitivity analysis and others	7	11.5%
Not specified	32	52.5%
Cost-effectiveness threshold		
WHO GDP/ GNI	21	34.4%
Woods et al. country estimates	1	1.64%
None	39	63.9%
Economic evaluation checklist		
Publications	61	
Maximum score	29	
Minimum score	6	
Mean score	20.9	
Standard Error	0.71	
Confidence Level (95%)	± 1.42	

The outcome measures were dominated by 31 studies that applied natural units such as life years gained or disease cases averted. The remaining 30 studies used DALYs, QALYs and a combination of DALY and natural units or a combination of DALYs and QALYs and natural units at proportions of 14.8%, 13.1%, 18%, 1.6% and 1.6%, respectively.

Time horizons of less than one year and those between one year and five years accounted for 62.2% of time horizons in all studies. Nine studies adopted time horizons of between 6 and 20 years; only six studies applied a lifetime horizon. Twenty-nine point five per cent of studies did not specify the discount rate used in their analysis, with most of these studies reporting a time horizon of less than one year. Twenty studies, accounting for 32.8% of all 61 included studies, used a 3% discount rate for both costs and benefits. A discount rate of 3% for costs and benefits, was applied in 13 and 7 studies, respectively. A discount rate of 5% was used in 3 studies, applying it only to costs in 2 studies and benefits in the remaining one study. The rationale for using the 3% discount rate includes that it is commonly used in sub-Saharan Africa, it has been suggested for standard calculations of DALYs, it is a common practice in economic evaluations, it has been used extensively in the literature, and the World Health Organisation recommends it. Two of the three studies reported the reason for using the 5% discount rate as the official discount rate used by the Ministry of Health in Zambia.

More than half of the studies did not specify the sensitivity analysis applied. Probabilistic sensitivity analysis was used as a standalone method in 10 studies and as combination with other techniques in 7 studies, constituting a total of 27.9% of studies that utilised probabilistic sensitivity analysis. Univariate, multivariate, and a combination of univariate and multivariate analyses accounted for 8.2%, 4.9%, and 6.6% of the methods used across studies, respectively. While not all studies reported the statistical tools used for analysis, some utilised Microsoft Excel, TreeAge, Stata, SAS, R, and MATLAB.

Only 22 of the 61 studies reported using a cost-effectiveness threshold in their analysis. Among these, 21 studies defined an intervention as 'highly cost-effective' if it averts a DALY for less than per capita gross domestic product (GDP) and as 'cost-effective' if it averts a DALY for less than three times per capita GDP. One study employed country-specific cost-effectiveness threshold estimates by Woods et al. of $68-$768 per gained in Zambia [91].

## Discussion

To our knowledge, this is the first systematic review to characterise the evidence for economic evaluation in Zambia. This review included 61 economic evaluation studies from 1993 to 2023. Results show an increase in research outputs between 2012 and 2023. Another important finding is the large number of non-local first authors and the small number of local authors across all studies. Findings from this review also show that a large portion of economic evaluation research has been conducted on infectious diseases, with HIV as the primary disease condition for the evaluations. The leading health technologies evaluated were program-based, and a health system perspective was predominantly applied. Most studies did not specify the model and the costing approach used for analysis; however, the data sources were characterised by trial-based data and a combination of program, published and unpublished data. Direct costs and natural unit measures were used in the analysis by applying a 3% discount rate for both costs and benefits. A critical omission in the included studies was a lack of specifications regarding the sensitivity analysis used. While most studies did not indicate the cost-effectiveness threshold used, those that did, referenced the WHO GDP threshold measure.

Compared to a similar Indian study, which identified 104 eligible publications, the present analysis only identified 61 published economic evaluations fulfilling the inclusion criteria [15]. However, this finding is comparable with other country-specific studiessuch as Nigeria (*n* = 44) [19], Ethiopia (*n* = 34) [20], Ghana (*n* = 31) [17], Zimbabwe (*n* = 26) [18], Ethiopia (*n* = 21) [21], Bangladesh (*n* = 12) [16], and Vietnam (*n* = 6) [14]. This observation is not surprising, as Zambia similarly ranked among the top four LMICs with significant economic evaluation studies according to a global review [11]. The study results show that all included studies listed their funding sources, including three that declared no funding. These results are similar to a review study in Bangladesh, where 83% of included studies stated their funding sources, but contrasts with South Africa, where as many as 45% of studies did not declare their funding sources [16, 92]. The Bill and Melinda Gates Foundation was identified as the lead funder for economic evaluation studies in Zambia. This observation is not unexpected, as the foundation dedicated over 200 million dollars between 2004 and 2015 to fund cost-effectiveness analysis programmes globally, including supporting the development of a reference case [93]. As identified elsewhere, financing from the private sector for economic evaluations was also low in this study, at 3% [11, 92]. A global review study by Pitt C and others suggests that private for-profit companies are less likely to be attracted to fund nonclinical research, which is a common demand in LMICs [11].

The production of economic evaluations in Zambia over the 30 years from 1993 was highest after 2012. We speculate that the launch of Zambia's Health Policy in 2012 and the National Health Care Package provided policy frameworks to advocate for the use of cost-effective health technologies as a means to support Zambia's universal health coverage agenda [23, 94]. Policy tools, such as Zambia’s 2017 to 2027 Health Care Financing Strategy and the National Health Strategic Plans for 2017 to 2021 and 2022 to 2026, have reinforced this further [22, 25, 95]. The production of economic evaluations in Zambia was led mainly by nonlocal first-authors from the United States of America or the United Kingdom, similar to other reviews [10, 16, 17]. The proportion of local-first authors at 10% was much lower than that reported in other similar country-specific studies at 30% [17], 26% [17] and 69% [92]. In addition, only 120 authors out of 563 were affiliated with an institution in Zambia, with the majority affiliated with the Ministry of Health’s National Malaria Control Centre. These results align with those established by a recent study on the status of the health economics workforce in Zambia, which identified limited local human resources and training programmes that might suggest the limited local leadership on economic evaluations [24]. As identified in the 2018 to 2024 Health Workforce Strategy and the Health Benefits Package Revision Road Map for Zambia, regional and international collaborations can strengthen local capacities [23, 96]. Future research collaborations should support more local involvement in economic evaluation research leadership to retain in-country capacity.

The results also show that studies were published in international journals, similar to a review in Ghana [17], but in contrast to South Africa [92], where almost half of the studies were published in local journals. This could be due to Zambia's lack of peer-reviewed and internationally indexed journals. Results from this study, identifying PLOS One as the journal with the most published studies, share similarities with a global review, that showed the journal ranked highly in publishing economic evaluation studies across LMICs [11].

Many studies could have stated whether they obtained ethical clearance from Zambia or a collaborating country. With the establishment of the Zambia National Health Research Authority, it is mandatory for all research conducted in Zambia to receive ethical clearance from the established authority [97]. In our review, 25% of the studies were multi-country, higher than the percentage reported in a similar review [92]. The concentration of research settings in Lusaka and the Southern Provinces can be explained by research collaboration sites such as Macha Research Trust and Lusaka’s status as the most densely populated province. The high density increases the likelihood to host programs and study sites for various organisations. However, future research should expand to more settings in the remaining eight provinces to align with Zambia's overreaching health policy objective of delivering cost-effective health services closer to the household [94].

The present systematic review has highlighted the dominance of cost-effectiveness studies as the preferred form of economic evaluation. This is consistent with other reviews [11, 16, 17]. The results also show that HIV, malaria, antenatal care, and Tuberculosis are leading conditions with economic evaluation evidence in Zambia. The results are aligned with the disease burden in Zambia which is characterised by high morbidity and mortality from these conditions, necessitating their classification as national health priorities [25]. Consequently, the country has been generating and implementing cost-effective disease-specific and system-wide interventions to eliminate the conditions as public health threats [25]. The starting point for prioritising disease control is currently not specified in the benefits revision roadmap for Zambia, [23]. 

Therefore, the identified availability of economic evaluation evidence on HIV, tuberculosis, malaria, and antenatal care presents an opportunity for consideration as a starting point for prioritisation. However, with non-communicable diseases accounting for 23% of total deaths in Zambia and being classified as a national health priority [25], future research could also support the availability of economic evaluation evidence in this priority area. Since it is not practically feasible to produce economic evaluation evidence for all disease areas needed for health benefits package prioritisation [7], methods to transfer and localise international economic evaluation, such as adaptive health technology assessment, can be considered [98].

The more extensive use of the health provider perspective in this study has also been observed elsewhere [21, 26]. The 39.3% results for studies that did not specify the type of perspective applied to their analysis were lower than those of a review in South Africa, 47% [92], and 85% in Bangladesh [16]. As seen in this study, other reviews have established the predominant use of trial-based data to support economic evaluations [10, 11, 17]. However, data availability challenges have also resulted in more studies combining trial-based, published and unpublished data. The dominant use of direct costs at 75.4% is lower than that reported in other studies, which extends to 100% [25, 26]. Ingredient-based costing was the leading approach in this study, with only one study combining the top-down and bottom-up approaches, unlike a similar review where this combination was observed more as a way to support accurate quantification of costs [12]. This study also identified decision trees and Markov modelling as leading tools, similar to another review [17].

The extensive use of natural units as outcome measures by the studies included in this review is similar to what has been identified by another review [16]. After natural units, the use of DALYs was high, identical to another review in which the use of DALYs was 48% [17]. Using DALYs is essential for donor-funded economic evaluation studies in low- and middle-income countries where cross-country comparisons are needed [10]. Our review also identified three studies that valued their outcomes using health-related quality-of-life measures. These measures could be challenging due to the difficulty and resources required to measure health status [16] and the lack of tools to derive DALYs and QALYs [99]. There is an increased call for more studies in LMICs to use quality-of-life measurements to capture health outcomes [10].

Other reviews, such as this one, have identified a more prominent use of time horizons, which are less than one year and between 1 and 5 years [17, 92]. Similarly, the 3% discount rate on costs and outcomes has been established in this study and elsewhere [16, 17]. However, authors must exercise caution when using the 3% social discount rate as it is inconsistent with the economic growth rates in LMICs and might result in overvaluing future costs and benefits [100]. Although half of the studies in this review could specify the sensitivity analysis applied in their analysis, as seen in another study [16], the dominant use of probabilistic sensitivity analysis identified is similar to that of other reviews [11, 17]. Incorporating sensitivity analysis into economic evaluations aids decision-making by illustrating how structural and parameter uncertainties impact the results [10]. As more analysis tools become available for economic evaluation, this study identified the growing use of the open-source software R, which increases transparency and transferability of analysis through the availability of analysis code, as advocated for in open science. In this review, 21 of the 22 studies applied the WHO-recommended GDP threshold measure, similar to other findings [10, 101]. Reporting cost-effectiveness thresholds would assist decision-makers in Zambia in assessing whether the cost for each benefit unit of a new intervention falls within the acceptable limits established by the health system for obtaining an additional benefit unit. Furthermore, as most of the cost-effectiveness studies did not use generic measures (QALYs and DALYs), it was challenging to judge effectiveness and the use of thresholds.

The study findings could support health benefits package reform policy by partially providing evidence to support the mapping, synthesis and appraisal roadmap stages. Additionally, this evidence could support researchers conducting economic evaluation research in Zambia on the status of available evidence, its characteristics and methods to inform a systematic and consistent economic evaluation approach in future research. The evidence gaps in sensitivity analysis, generic quality of life measurements, and thresholds could indicate a potential capacity gap in Zambia that needs strengthening [10].These results are also crucial for the local institutional review boards as a reference tool to ensure that the evidence and conduct of economic evaluation evidence in Zambia are consistent with the country’s research needs and methods gaps. These results could form background evidence for policy-makers to support the decision-making process for developing HTA guidelines for Zambia.

### Limitations

This review had limitations. Although some findings from this study fit into discussions on economic evaluations in LMICs, they are unique only to Zambia, and caution must be applied to their generalisation to settings other than Zambia. The study restrictions imposed by the study can potentially exclude publications, although necessary attempts were made to ensure all relevant studies were assessed for eligibility. Furthermore, this study did not evaluate the quality of the included studies. Therefore, future research should gather data regarding the quality of the cost-effectiveness analyses or the reported incremental cost-effectiveness ratios to accurately convey the risks pertinent to decision-making for the benefits package design process's appraisal committee.

## Conclusions

This study is the first attempt to characterise economic evaluation evidence in Zambia. The study results add to the growing body of evidence in sub-Saharan Africa that shows low first authorship status for local researchers, limited local research funding, some inconsistencies in methods, and concentration of research on infectious diseases such as HIV. The findings on the quantity and conduct of economic evaluations form a part of the evidence necessary to support the evidence appraisal phase for Zambia's benefits package revision process. It also provides a reference source for researchers and decision-makersto increase transparency, consistency, and methodological quality in economic evaluation research in Zambia.

## Supplementary Information

Supplementary Material 1.

Supplementary Material 2.

Supplementary Material 3.

## Data Availability

All data relevant to the study are included in the article or uploaded as supplementary information. The protocol for this systematic review is not publicly available but can be accessed from the corresponding author.
