# Supplementary material for: Health technology assessment to support health benefits package design: a systematic review of economic evaluation evidence in Zambia
Source: BMC Health Serv Res. 2024 Nov 18;24:1426. doi: 10.1186/s12913-024-11914-z (PMC11572362; doi:10.1186/s12913-024-11914-z)
Supplement: Supplementary file 2 — Supplementary Material 2. [file 12913_2024_11914_MOESM2_ESM.docx]

**Supplementary File 2: Search terms and PubMed search strategy**

**Section 1: Search Terms**

| **Categories** | **Key terms** | **Words and phrases** |
| --- | --- | --- |
| Economic evaluation | Economic evaluation, cost-utility, cost-benefit, cost-effectiveness | "cost benefit analysis"[MeSH Terms] OR ("cost benefit"[All Fields] AND "analysis"[All Fields]) OR "cost benefit analysis"[All Fields] OR ("economic"[All Fields] AND "evaluation"[All Fields]) OR "economic evaluation"[All Fields] OR "cost-utility"[All Fields] OR ("cost benefit analysis"[MeSH Terms] OR ("cost benefit"[All Fields] AND "analysis"[All Fields]) OR "cost benefit analysis"[All Fields] OR ("cost"[All Fields] AND "benefit"[All Fields]) OR "cost benefit"[All Fields]) OR ("cost effectiveness analysis"[MeSH Terms] OR ("cost effectiveness"[All Fields] AND "analysis"[All Fields]) OR "cost effectiveness analysis"[All Fields] OR ("cost"[All Fields] AND "effectiveness"[All Fields]) OR "cost effectiveness"[All Fields]) |
| Disease control priority | Diseases, Communicable diseases, Neglected tropical diseases, Foundations of care, Growth, development and ageing, Noncommunicable diseases and mental health, Pregnancy and birth, Sexual health, Violence and injury | "disease"[MeSH Terms] OR "disease"[All Fields] OR "diseases"[All Fields] OR "disease s"[All Fields] OR "diseased"[All Fields] OR "Neglected tropical diseases"[All Fields] OR "growth development and ageing"[All Fields] OR "Noncommunicable diseases"[All Fields] OR ("mental health"[MeSH Terms] OR ("mental"[All Fields] AND "health"[All Fields]) OR "mental health"[All Fields]) OR "Pregnancy and birth"[All Fields] OR ("sexual health"[MeSH Terms] OR ("sexual"[All Fields] AND "health"[All Fields]) OR "sexual health"[All Fields]) OR "Violence and injury"[All Fields] |
| Health technology | Health technology, medical device, diagnostic and equipment, laboratory and laboratory equipment, screening, interventions, programs, pharmaceuticals, health system | "Health technology"[All Fields] OR "medical device"[All Fields] OR ("diagnosis"[MeSH Terms] OR "diagnosis"[All Fields] OR "diagnostic"[All Fields] OR "diagnostical"[All Fields] OR "diagnostically"[All Fields] OR "diagnostics"[All Fields]) OR ("laboratorial"[All Fields] OR "laboratories"[MeSH Terms] OR "laboratories"[All Fields] OR "laboratory"[All Fields] OR "laboratory s"[All Fields]) OR ("diagnosis"[MeSH Subheading] OR "diagnosis"[All Fields] OR "screening"[All Fields] OR "mass screening"[MeSH Terms] OR ("mass"[All Fields] AND "screening"[All Fields]) OR "mass screening"[All Fields] OR "early detection of cancer"[MeSH Terms] OR ("early"[All Fields] AND "detection"[All Fields] AND "cancer"[All Fields]) OR "early detection of cancer"[All Fields] OR "screen"[All Fields] OR "screenings"[All Fields] OR "screened"[All Fields] OR "screens"[All Fields]) OR ("intervention s"[All Fields] OR "interventions"[All Fields] OR "interventive"[All Fields] OR "methods"[MeSH Terms] OR "methods"[All Fields] OR "intervention"[All Fields] OR "interventional"[All Fields]) OR "program"[All Fields] OR ("drug s"[All Fields] OR "pharmaceutical preparations"[MeSH Terms] OR ("pharmaceutical"[All Fields] AND "preparations"[All Fields]) OR "pharmaceutical preparations"[All Fields] OR "drugs"[All Fields]) OR ("medicin"[All Fields] OR "medicinal"[All Fields] OR "medicinally"[All Fields] OR "medicinals"[All Fields] OR "medicine"[MeSH Terms] OR "medicine"[All Fields] OR "medicine s"[All Fields] OR "medicines"[All Fields]) OR "health system"[All Fields] |
| Restrictions | Zambia, Humans, NOT animals, | (("zambia"[MeSH Terms] OR "zambia"[All Fields] OR "zambia s"[All Fields]) AND ("human s"[All Fields] OR "humans"[MeSH Terms] OR "humans"[All Fields] OR "human"[All Fields])) NOT ("animals"[MeSH Terms:noexp] NOT "animal"[All Fields]) |

**Section 2: Search Strategy**

| **PubMed search strategy** | ((("zambia"[MeSH Terms] OR "zambia"[All Fields] OR "zambia s"[All Fields]) AND ("human s"[All Fields] OR "humans"[MeSH Terms] OR "humans"[All Fields] OR "human"[All Fields])) NOT ("animals"[MeSH Terms:noexp] NOT "animal"[All Fields])) AND ("Health technology"[All Fields] OR "medical device"[All Fields] OR ("diagnosis"[MeSH Terms] OR "diagnosis"[All Fields] OR "diagnostic"[All Fields] OR "diagnostical"[All Fields] OR "diagnostically"[All Fields] OR "diagnostics"[All Fields]) OR ("laboratorial"[All Fields] OR "laboratories"[MeSH Terms] OR "laboratories"[All Fields] OR "laboratory"[All Fields] OR "laboratory s"[All Fields]) OR ("diagnosis"[MeSH Subheading] OR "diagnosis"[All Fields] OR "screening"[All Fields] OR "mass screening"[MeSH Terms] OR ("mass"[All Fields] AND "screening"[All Fields]) OR "mass screening"[All Fields] OR "early detection of cancer"[MeSH Terms] OR ("early"[All Fields] AND "detection"[All Fields] AND "cancer"[All Fields]) OR "early detection of cancer"[All Fields] OR "screen"[All Fields] OR "screenings"[All Fields] OR "screened"[All Fields] OR "screens"[All Fields]) OR ("intervention s"[All Fields] OR "interventions"[All Fields] OR "interventive"[All Fields] OR "methods"[MeSH Terms] OR "methods"[All Fields] OR "intervention"[All Fields] OR "interventional"[All Fields]) OR "program"[All Fields] OR ("drug s"[All Fields] OR "pharmaceutical preparations"[MeSH Terms] OR ("pharmaceutical"[All Fields] AND "preparations"[All Fields]) OR "pharmaceutical preparations"[All Fields] OR "drugs"[All Fields]) OR ("medicin"[All Fields] OR "medicinal"[All Fields] OR "medicinally"[All Fields] OR "medicinals"[All Fields] OR "medicine"[MeSH Terms] OR "medicine"[All Fields] OR "medicine s"[All Fields] OR "medicines"[All Fields]) OR "health system"[All Fields]) AND ("disease"[MeSH Terms] OR "disease"[All Fields] OR "diseases"[All Fields] OR "disease s"[All Fields] OR "diseased"[All Fields] OR "Neglected tropical diseases"[All Fields] OR "growth development and ageing"[All Fields] OR "Noncommunicable diseases"[All Fields] OR ("mental health"[MeSH Terms] OR ("mental"[All Fields] AND "health"[All Fields]) OR "mental health"[All Fields]) OR "Pregnancy and birth"[All Fields] OR ("sexual health"[MeSH Terms] OR ("sexual"[All Fields] AND "health"[All Fields]) OR "sexual health"[All Fields]) OR "Violence and injury"[All Fields]) AND ("disease"[MeSH Terms] OR "disease"[All Fields] OR "diseases"[All Fields] OR "disease s"[All Fields] OR "diseased"[All Fields] OR "Neglected tropical diseases"[All Fields] OR "growth development and ageing"[All Fields] OR "Noncommunicable diseases"[All Fields] OR ("mental health"[MeSH Terms] OR ("mental"[All Fields] AND "health"[All Fields]) OR "mental health"[All Fields]) OR "Pregnancy and birth"[All Fields] OR ("sexual health"[MeSH Terms] OR ("sexual"[All Fields] AND "health"[All Fields]) OR "sexual health"[All Fields]) OR "Violence and injury"[All Fields]) AND ("cost benefit analysis"[MeSH Terms] OR ("cost benefit"[All Fields] AND "analysis"[All Fields]) OR "cost benefit analysis"[All Fields] OR ("economic"[All Fields] AND "evaluation"[All Fields]) OR "economic evaluation"[All Fields] OR "cost-utility"[All Fields] OR ("cost benefit analysis"[MeSH Terms] OR ("cost benefit"[All Fields] AND "analysis"[All Fields]) OR "cost benefit analysis"[All Fields] OR ("cost"[All Fields] AND "benefit"[All Fields]) OR "cost benefit"[All Fields]) OR ("cost effectiveness analysis"[MeSH Terms] OR ("cost effectiveness"[All Fields] AND "analysis"[All Fields]) OR "cost effectiveness analysis"[All Fields] OR ("cost"[All Fields] AND "effectiveness"[All Fields]) OR "cost effectiveness"[All Fields])) |
| --- | --- |
