# Supplementary material for: Health technology assessment to support health benefits package design: a systematic review of economic evaluation evidence in Zambia
Source: BMC Health Serv Res. 2024 Nov 18;24:1426. doi: 10.1186/s12913-024-11914-z (PMC11572362; doi:10.1186/s12913-024-11914-z)
Supplement: Supplementary file 3 — Supplementary Material 3. [file 12913_2024_11914_MOESM3_ESM.docx]

**Supplementary File 3: Selected background characteristics of included studies**

| **Article** | **Country of First Author** | **Sources of Funding** | **Institution of First Author** | **Local-only authors** | **Publishing Journal** | **Type of intervention** | **Form of Economic valuation** |
| --- | --- | --- | --- | --- | --- | --- | --- |
| Gray RH et al. (1993). Evaluation of natural family planning programmes in Liberia and Zambia | United States of America | United States Agency for International Development; Georgetown University; Johns Hopkins University | Johns Hopkins University | 1 | Journal of Biosocial Science | Natural family planning | Cost effectiveness analysis |
| Foster S et al. (1995). Benefits of HIV screening of blood transfusions in Zambia | United Kingdom | United Kingdom Overseas Development Administration | London School of Hygiene and Tropical Medicine | 0 | Lancet | Bloods Screening for HIV | Cost benefit analysis |
| Foster S et al. (1997). Modelling the economic benefits of tuberculosis preventive therapy for people with HIV: The example of Zambia | United Kingdom | United Kingdom Overseas Development Administration | London School of Hygiene and Tropical Medicine | 1 | AIDS | TB preventive therapy programme using daily isoniazid for 6 months | Cost benefit analysis |
| Walker D et al. (2000). An incremental cost-effectiveness analysis of the first, second and third sputum examination in the diagnosis of pulmonary tuberculosis | United Kingdom | Department for International Development | London School of Hygiene and Tropical Medicine | 1 | International Journal of Tuberculosis and Lung Diseases | Screening for pulmonary tuberculosis; serial diagnosis at first, second and third sputum | Cost effectiveness analysis |
| Utzinger J et al. (2001). Efficacy and cost-effectiveness of environmental management for malaria control | United States of America | Swiss National Science Foundation | Princeton University | 0 | Tropical Medicine and International Health | Environmental management for malaria control | Cost effectiveness analysis |
| Utzinger J et al. (2002). The economic payoffs of integrated malaria control in the Zambian copperbelt between 1930 and 1950 | United States of America | Princeton University; Swiss National Science Foundation | Princeton University, Princeton | 0 | Tropical Medicine and International Health | integrated malaria control | Cost effectiveness analysis |
| Stringer EM et al. (2003). Prevention of mother-to-child transmission of HIV in Africa: Successes and challenges in scaling-up a nevirapine-based program in Lusaka, Zambia | USA; Zambia | National Institutes of Health | University of Alabama at Birmingham; University of Zambia; Centre for Infectious Disease Research in Zambia | 4 | AIDS | Nevirapine-based Prevention of mother-to-child transmission of HIV | Cost effectiveness analysis |
| Sweat MD et al. (2004). Cost-effectiveness of nevirapine to prevent mother-to-child HIV transmission in eight African countries | United States of America | World Health Organization; United States National Institute of Mental Health | Johns Hopkins University | 0 | AIDS | Nevirapine to prevent mother-to- child HIV transmission | Cost effectiveness analysis |
| Fullerton JT et al. (2006). Active management of third stage of labour saves facility costs in Guatemala and Zambia | United States of America | United States Agency for International Development | Project Concern International | 0 | Journal of Health population Nutrition | Active Management of Third Stage Labour | Cost benefit analysis |
| Chi BH et al. (2006). Field performance of a thin-layer chromatography assay for detection of nevirapine in umbilical cord blood | USA; Zambia | Elizabeth Glaser Paediatric AIDS Foundation; United States National Institutes of Health; Boehringer- Ingelheim, Inc | Centre for Infectious Disease Research in Zambia; University of Alabama | 2 | HIV Clinical Trials | Field Performance of a Thin-Layer Chromatography Assay for Detection of Nevirapine in PMTCT | Cost effectiveness analysis |
| Chanda P et al. (2007). A cost-effectiveness analysis of artemether lumefantrine for treatment of uncomplicated malaria in Zambia | Zambia | Novartis Pharmaceuticals | National Malaria Control Centre | 6 | Malaria Journal | Artemether lumefantrine for treatment of uncomplicated malaria | Cost effectiveness analysis |
| Ryan M et al. (2008). The cost-effectiveness of cotrimoxazole prophylaxis in HIV-infected children in Zambia | Ireland | Advisory Board of Irish Aid | Trinity College, Dublin | 4 | AIDS | Cotrimoxazole prophylaxis in HIV-infected children older than 12 months | Cost effectiveness analysis |
| Mueller DH et al. (2008). Costs and cost-effectiveness of tuberculosis cultures using solid and liquid media in a developing country | United Kingdom | FIND; United Kingdom Department for International Development; Johns Hopkins University; Bill and Melinda Gates Foundation | London School of Hygiene & Tropical Medicine | 4 | The International Journal of Tuberculosis and Lung Disease | Tuberculosis cultures using solid and liquid media | Cost effectiveness analysis |
| Chanda P et al. (2009). Cost-effectiveness analysis of the available strategies for diagnosing malaria in outpatient clinics in Zambia | Zambia | Swedish International Development Aid; Novartis Pharma | National Malaria Control Centre | 2 | Cost Effectiveness and Resource Allocation | Diagnosing malaria in outpatient clinics using Clinical, microscopy and RDTs | Cost effectiveness analysis |
| Bachmann MO (2009). Cost effectiveness of community-based therapeutic care for children with severe acute malnutrition in Zambia: decision tree model | United Kingdom | Valid International and Concern | University of East Anglia | 1 | Cost Effectiveness and Resource Allocation | Community-based therapeutic care for children with severe acute malnutrition | Cost effectiveness analysis |
| Chanda P et al. (2011). Relative costs and effectiveness of treating uncomplicated malaria in two rural districts in Zambia: Implications for nationwide scale-up of home-based management | Zambia | World Health Organization/Tropical Disease Research | Ministry of Health | 5 | Malaria Journal | Managing uncomplicated malaria with ACT and RDTs using CHWs in comparison with the health facility model. | Cost effectiveness analysis |
| Manasyan A et al. (2011). Cost-effectiveness of Essential Newborn Care Training in Urban First-Level Facilities | United States of America | Eunice Kennedy Shriver National Institute of Child Health and Human Development; Global Network for Women’s and Children’s Health Research; Bill and Melinda Gates Foundation | University of Alabama, | 0 | The American Academy of Paediatrics | Essential Newborn Care Training in Urban First-Level Facilities | Cost effectiveness analysis |
| Njeuhmeli E et al. (2011). Voluntary medical male circumcision: modeling the impact and cost of expanding male circumcision for HIV prevention in eastern and southern Africa | United States of America | The United States President's Emergency Plan For AIDS Relief; United States Agency for International Development; The Joint United Nations Programme on HIV and AIDS | United States Agency for International Development | 0 | PloS Medicine | Voluntary Medical Male Circumcision for HIV Prevention | Cost effectiveness analysis |
| Marseille E et al. (2012). Taking ART to Scale: Determinants of the Cost and Cost-Effectiveness of Antiretroviral Therapy in 45 Clinical Sites in Zambia | United States of America | U.S. Center for Disease Control and Prevention | Health Strategies International | 5 | PloS One | Scale of Antiretroviral Therapy for HIV management | Cost effectiveness analysis |
| Sabin LL et al. (2012). Costs and cost-effectiveness of training traditional birth attendants to reduce neonatal mortality in the lufwanyama neonatal survival study (LUNESP) | United States of America | United States Agency for International Development; American Academy of Pediatrics; UNICEF | Boston University | 2 | PloS One | Training Traditional Birth Attendants to Reduce Neonatal Mortality | Cost effectiveness analysis |
| Sedlmayr R et al. (2013). Health impact and cost-effectiveness of a private sector bed net distribution: experimental evidence from Zambia | United States of America | Bill and Melinda Gates Foundation; Malaria Control and Elimination Partnership in Africa | No Institution | 3 | Malaria Journal | Private sector (ITN) bed net distribution | Cost effectiveness analysis |
| Jeffrey E et al. (2013). Health benefits, costs, and cost-effectiveness of earlier eligibility for adult antiretroviral therapy and expanded treatment coverage: a combined analysis of 12 mathematical models | United Kingdom | The Bill and Melinda Gates Foundation; World Health Organization | Imperial College London | 0 | Lancet Glob Health. | Eligibility for adult antiretroviral therapy and expanded treatment coverage | Cost effectiveness analysis |
| Stanback J et al. (2013). Does free pregnancy testing reduce service denial in family planning clinics? A cluster-randomized experiment in Zambia and Ghana | United States of America | United States Agency for International Development | FHI 360 | 2 | Global Health: Science and Practice | Pregnancy testing | Cost effectiveness analysis |
| Nichols BE et al (2013). Cost-Effectiveness of Pre-Exposure Prophylaxis (PrEP) in Preventing HIV-1 Infections in Rural Zambia: A Modeling Study | Netherlands | ids Fonds Netherlands; European Union | Erasmus Medical Centre | 1 | PloS One | Pre-Exposure Prophylaxis (PrEP) in Preventing HIV-1 Infections with daily oral tenofovir and emtricitabine | Cost effectiveness analysis |
| Fiedler JL et al. (2013). Assessing Zambia's industrial fortification options: Getting beyond changes in prevalence and cost-effectiveness | United States of America | None indicated | international Food Policy Research Institute | 1 | Food and Nutrition Bulletin | Nutrient fortification to support Growth, development and ageing | Cost effectiveness analysis |
| Sikaala CH et al. (2014). A cost-effective, community-based, mosquito-trapping scheme that captures spatial and temporal heterogeneities of malaria transmission in rural Zambia | Zambia; UK | Bill & Melinda Gates Foundation | National Malaria Control Centre; Liverpool School of Tropical Medicine, | 5 | Malaria Journal | Community-based, mosquito- trapping scheme | Cost effectiveness analysis |
| Larson BA et al (2014) . Finding a Needle in the Haystack: The costs and cost-effectiveness of syphilis diagnosis and treatment during pregnancy to prevent congenital syphilis in Kalomo District of Zambia | United States of America | President’s Emergency Plan for AIDS Relief (PEPFAR); through the Centers for Disease Control and Prevention | Boston University | 1 | PloS One | Syphilis Diagnosis and Treatment during Pregnancy | Cost effectiveness analysis |
| Nichols BE et al (2014). Cost-effectiveness of PrEP in HIV/AIDS control in Zambia: A stochastic league approach | Netherlands | AIDS Fonds Netherlands; European Union | Erasmus Medical Centre | 2 | Journal of Acquired Immune Defic Syndr | Antiretroviral therapy initiation and pre-exposure prophylaxis to prevent HIV | Cost effectiveness analysis |
| Koethe JR et al (2014). Estimating the cost-effectiveness of nutrition supplementation for malnourished, HIV-infected adults starting antiretroviral therapy in a resource-constrained setting | USA; Zambia | National Institutes of Health/National Institute of Allergy and Infectious Diseases; Fogarty International Center | Centre for Infectious Diseases Research in Zambia; Vanderbilt University School of Medicine | 0 | Cost Effectiveness and Resource Allocation | Nutrition supplementation for malnourished, HIV-infected adults starting antiretroviral therapy | Cost effectiveness analysis |
| Ishikawa N et al. (2014). Health outcomes and cost impact of the new WHO 2013 Guidelines on prevention of mother-to-child transmission of HIV in Zambia | Japan | Ministry of Health, Labour and Welfare, Japan | National Center for Global Health and Medicine | 3 | PloS One | Prevention of Mother-to-Child Transmission of HIV | Cost effectiveness analysis |
| Griffiths UK et al. (2014). Cost-effectiveness of eye care services in Zambia | United Kingdom | Standard Chartered Bank; Sight savers; Christian Blind Mission. | London School of Hygiene & Tropical Medicine | 1 | Cost Effectiveness and Resource Allocation | Cataract surgery and refractive error/presbyopia correction | Cost effectiveness analysis |
| Gopalappa C et al. (2014). The costs and benefits of Option B+ for the prevention of mother-to-child transmission of HIV | United States of America | Bill and Melinda Gates Foundation | Futures Institute | 0 | AIDS | Option B+ for the prevention of mother-to-child transmission of HIV | Cost effectiveness analysis |
| Silumbe K et al. (2015). Costs and cost-effectiveness of a large-scale mass testing and treatment intervention for malaria in Southern Province, Zambia | Zambia | Bill & Melinda Gates Foundation through the PATH-MACEPA program | Malaria Control and Evaluation Partnership in Africa | 4 | Malaria Journal | Large-scale mass testing and treatment for malaria | Cost effectiveness analysis |
| Downing J et al (2015) . Cost-effectiveness of the non-pneumatic anti-shock garment (NASG): evidence from a cluster randomized controlled trial in Zambia and Zimbabwe | United States of America | Bill & Melinda Gates Foundation | University of California | 1 | BMC Health Services Research | Non-pneumatic anti-shock garment for post-partum haemorrhage | Cost effectiveness analysis |
| Award SF et al. (2015) . Investigating Voluntary Medical Male Circumcision Program Efficiency Gains through Subpopulation Prioritization: Insights from Application to Zambia | QATAR | Bill & Melinda Gates Foundation | Weill Cornell Medical College | 1 | PloS One | Voluntary Medical Male Circumcision Program Efficiency Gains through Subpopulation Prioritization | Cost effectiveness analysis |
| Lividini K et al. (2015) . Assessing the promise of biofortification: A case study of high provitamin A maize in Zambia | United States of America | Bill & Melinda Gates Foundation | International Food Policy Research Institute | 0 | food policy | Biofortification for micronutrient for vitamin A | Cost effectiveness analysis |
| Terris-Prestholt F. et al. (2015). The cost-effectiveness of 10 antenatal syphilis screening and treatment approaches in Peru, Tanzania, and Zambia | United Kingdom | Bill and Melinda Gates Foundation; WHO | London School of Hygiene and Tropical Medicine | 0 | International Journal of Gynaecology and Obstetrics | Antenatal syphilis screening and treatment approaches | Cost effectiveness analysis |
| Shelley KD et al. (2015). Scaling down to scale up: A health economic analysis of integrating point-of-care syphilis testing into antenatal care in Zambia during pilot and national rollout implementation | United States of America | Bill & Melinda Gates Foundation through UNICEF; UNDP; World Bank; World Health Organization | George Washington University | 2 | PloS One | Point-of-Care Syphilis Testing for the prevention of mother-to- child-transmission of HIV programmes | Cost effectiveness analysis |
| Linde M et al. (2015). Cost-effectiveness analysis of interventions for migraine in four low- and middle-income countries | Norway | No funding | Norwegian University of Science and Technology | 0 | The Journal of Headache and Pain | First-line acute and prophylactic drugs, consumer education and provider training strategies for migraine management | Cost effectiveness analysis |
| Hewett PC et al. (2016). Randomized evaluation and cost-effectiveness of HIV and sexual and reproductive health service referral and linkage models in Zambia | United States of America | United States Agency for International Development | Population Council, | 2 | BMC Public Health | HIV and sexual and reproductive health service referral and linkage models | Cost effectiveness analysis |
| Wang P et al. (2016). Measuring the impact of non-monetary incentives on facility delivery in rural Zambia: a clustered randomised controlled trial | Zambia | Department for International Development | Idinsight | 4 | Tropical Medicine and International Health | Non-monetary incentives on facility delivery in rural | Cost effectiveness analysis |
| Shinsuke M et al. (2017). Cost-effectiveness analysis of the national decentralization policy of antiretroviral treatment programme in Zambia | Japan | National Center for Global Health and Medicine | National Center for Global Health and Medicine | 1 | Cost Effectiveness and Resource Allocation | Decentralization policy of antiretroviral treatment | Cost effectiveness analysis |
| Zeng W et al. (2018). Cost-effectiveness of results-based financing, Zambia: a cluster randomized trial | United States of America | World Bank through the Health Results Innovation Trust Fund | Brandeis University, | 0 | Bull World Health Organ | Results-based financing for RMNCH | Cost effectiveness analysis |
| Chen T et al. (2019). Healthcare Costs and Life-years Gained from Treatments Within the Advancing Cryptococcal Meningitis Treatment for Africa (ACTA) Trial on Cryptococcal Meningitis: A Comparison of Antifungal Induction Strategies in Sub-Saharan Africa | United Kingdom | Medical Research Council in the United Kingdom; French Agency for Research on AIDS; Viral Hepatitis; Wellcome Trust UK | Liverpool School of Tropical Medicine | 4 | Clinical Infectious Diseases | Antifungal Induction Strategies for Cryptococcal Meningitis Treatment | Cost effectiveness analysis |
| Pooran A et al. (2019). Point of care Xpert MTB/RIF versus smear microscopy for tuberculosis diagnosis in southern African primary care clinics: a multicentre economic evaluation | South Africa | European Union European; Developing Countries Clinical Trials Partnership; South African Medical Research Council. | UCT | 2 | Lancet Glob Health | Xpert MTB/RIF versus smear microscopy for tuberculosis diagnosis  tuberculosis diagnosis | Cost effectiveness analysis |
| Gershon N et al (2019). Cost effectiveness and affordability of trastuzumab in sub-Saharan Africa for early stage HER2-positive breast cancer | Israel | No funding | Ben-Gurion University of the Negev | 0 | Cost Effectiveness and Resource Allocation | Use of trastuzumab for breast cancer management | Cost effectiveness analysis |
| Wall KM et al. (2019). HIV testing and counselling couples together for affordable HIV prevention in Africa | United States of America | Canadian Government through Foreign Affairs, Trade and Development Canada; United Kingdom Department for International Development; AIDS Vaccine Initiative; United States Agency for International Development; AIDS International Training and Research Program Fogarty International Center; Fogarty International Center; Centers for Disease Control ; | Emory University | 1 | International Journal of Epidemiology | HIV testing and counselling for couples together strategy | Cost effectiveness analysis |
| Johns B et al. (2019). The costs and cost-effectiveness of a district-strengthening strategy to mitigate the 3 delays to quality maternal health care: Results from Uganda and Zambia | United States of America | USAID; US Centers for Disease Control and Prevention; Merck for Mothers | Abt Associates Inc | 3 | Global Health: Science and Practice | Strategy to mitigate the 3 delays to quality maternal health care | Cost effectiveness analysis |
| Tembo T et al. (2019). Evaluating the costs of cholera illness and cost-effectiveness of a single dose oral vaccination campaign in Lusaka, Zambia | Zambia | Centre for Infectious Disease Research in Zambia; Agence de Me ́decine Pre ́ventive | Centre for Infectious Disease Research in Zambia | 8 | PloS One | Oral cholera vaccination campaign and illness treatment with single dose | Cost effectiveness analysis |
| Anderson JD et al. (2019). Heterogeneity in potential impact and cost-effectiveness of ETEC and Shigella vaccination in four sub-Saharan African countries | United States of America | PATH from the Bill & Melinda Gates Foundation | Appalachian State University | 0 | Vaccine: X | ETEC and Shigella vaccine/ drug for dirhera impact on death and stunting | Cost effectiveness analysis |
| Yukich JO et al. (2020). Cost-Effectiveness of Focal Mass Drug Administration and Mass Drug Administration with Dihydroartemisinin-Piperaquine for Malaria Prevention in Southern Province, Zambia: Results of a Community-Randomized Controlled Trial | United States of America | Bill & Melinda Gates Foundation | Tulane University School of Public Health and Tropical Medicine | 2 | The American Society of Tropical Medicine and Hygiene | Mass drug admi to Dihydroartemisinic–Piperaquine for Malaria Prevention | Cost effectiveness analysis |
| Wall KM et al. (2020). Cost-effectiveness of integrated HIV prevention and family planning services for Zambian couples | United States of America | DFID | Emory University | 0 | AIDS | Voluntary HIV counselling and testing and couples’ family planning counselling strategies | Cost effectiveness analysis |
| Pretorius C et al.(2020). Modelling impact and cost-effectiveness of oral pre-exposure prophylaxis in 13 low-resource countries | United States of America | United States President’s Emergency Plan for AIDS Relief; United States Agency for International Development | Avenir Health | 0 | Journal of the International AIDS Society | Oral pre-exposure prophylaxis impact | Cost effectiveness analysis |
| Youngji J et al. (2021). Costs and cost-effectiveness of a comprehensive tuberculosis case finding strategy in Zambia | United States of America | Global Affairs Canada; Bill and Melinda Gates Foundation, Korea Health Industry Development Institute; Ministry of Health and Welfare, Republic of Korea | Johns Hopkins Bloomberg | 4 | PloS One | Comprehensive tuberculosis case finding strategy | Cost effectiveness analysis |
| Ranjeeta T et al(2021). Cost and cost-effectiveness of a universal HIV testing and treatment intervention in Zambia and South Africa: evidence and projections from the HPTN 071 (PopART) trial | United Kingdom | National Institutes of Health; President’s Emergency Plan for AIDS Relief; International Initiative for Impact Evaluation; Bill & Melinda Gates Foundation | London School of Economics and Political Science | 6 | Lancet Global health | Universal HIV testing and treatment intervention | Cost effectiveness analysis |
| Yang Y et al. (2021). Cost-Effectiveness Comparison of the ReMiND program and the NewHints Program for Reducing Neonatal Mortality Rates in the Muchinga Province of Zambia | United States of America | None indicated | Georgetown University | 0 | Public Health Review | ReMiNDand the NewHints Program for Reducing Neonatal Mortality Rates | Cost effectiveness analysis |
| Broucker GD et al. (2021). The cost-effectiveness of scaling-up rapid point-of-care testing for early infant diagnosis of HIV in southern Zambia | United States of America | National Institutes of Allergy and Infectious Disease | Johns Hopkins Bloomberg School of Public Health | 4 | PloS One | Rapid point-of-care testing for early infant diagnosis of HIV PMTCT | Cost effectiveness analysis |
| Ferrari G et al. (2022). Prevention of violence against women and girls: A cost-effectiveness study across 6 low- and middle-income countries | United Kingdom | UK AID through South African Medical Research Council | London School of Economics and Political Science; London School of Hygiene & Tropical Medicine; University of Bristol, Bristol Medical School | 1 | PLOS Medicine | Prevention strategies of violence against women and girls | Cost effectiveness analysis |
| Mtalimanja M et al. (2022). Economic evaluation of severe malaria in children under 14 years in Zambia | China | No funding | China Pharmaceutical University | 1 | Cost Effectiveness and Resource Allocation | Use of rtesunate against quinine in severe malaria in children under 14 years | Cost effectiveness analysis |
| Yukich J et al. (2022). Incremental cost and cost-effectiveness of the addition of indoor residual spraying with pirimiphos-methyl in sub-Saharan Africa versus standard malaria control: results of data collection and analysis in the Next Generation Indoor Residual Sprays (NgenIRS) project, an economic-evaluation | United Kingdom | Unitaid | Tropical Health Consulting | 6 | Malaria Journal | Indoor residual spraying with pirimiphos-methyl (Actellic) | Cost effectiveness analysis |
| Chisanga B et al. (2023). The economic impacts of house screening against malaria transmission: Experimental evidence from eastern Zambia | Netherlands | orld Health Organization Regional Office for Africa; Swedish International Development Cooperation Agency; Swiss Agency for Development and Cooperation; Australian Centre for International Agricultural Research; Federal Democratic Republic of Ethiopia; and the Gov- ernment of the Republic of Kenya | Wageningen University | 1 | House screening against malaria transmission | Cost benefit analysis |  |
